# Using an Interpretable Amino Acid-Based Machine Learning Method to Enhance the Diagnosis of Major Depressive Disorder

**DOI:** 10.3390/jcm13051222

**Published:** 2024-02-21

**Authors:** Cyrus Su Hui Ho, Trevor Wei Kiat Tan, Howard Cai Hao Khoe, Yee Ling Chan, Gabrielle Wann Nii Tay, Tong Boon Tang

**Affiliations:** 1 Department of Psychological Medicine, Yong Loo Lin School of Medicine, National University of Singapore, Singapore 117543, Singapore; gtwn@nus.edu.sg; 2 Centre for Sleep and Cognition, Yong Loo Lin School of Medicine, National University of Singapore, Singapore 117543, Singapore; trevor.tan@u.nus.edu; 3 Centre for Translational MR Research, Yong Loo Lin School of Medicine, National University of Singapore, Singapore 117543, Singapore; 4 Department of Electrical and Computer Engineering, National University of Singapore, Singapore 117583, Singapore; 5 N.1 Institute for Health & Institute for Digital Medicine (WisDM), National University of Singapore, Singapore 117456, Singapore; 6 Integrative Sciences and Engineering Programme (ISEP), National University of Singapore, Singapore 119077, Singapore; 7 Singapore Psychiatry Residency, National Healthcare Group, Singapore 308433, Singapore; howard.khoe@mohh.com.sg; 8 Centre for Intelligent Signal and Imaging Research (CISIR), Universiti Teknologi PETRONAS (UTP), Seri Iskandar 32610, Perak, Malaysia; yee_20001812@utp.edu.my (Y.L.C.); tongboon.tang@utp.edu.my (T.B.T.)

**Keywords:** major depressive disorder, diagnosis, biomarker, metabolomics, amino acids, machine learning

## Abstract

**Background:** Major depressive disorder (MDD) is a leading cause of disability worldwide. At present, however, there are no established biomarkers that have been validated for diagnosing and treating MDD. This study sought to assess the diagnostic and predictive potential of the differences in serum amino acid concentration levels between MDD patients and healthy controls (HCs), integrating them into interpretable machine learning models. **Methods:** In total, 70 MDD patients and 70 HCs matched in age, gender, and ethnicity were recruited for the study. Serum amino acid profiling was conducted by means of chromatography-mass spectrometry. A total of 21 metabolites were analysed, with 17 from a preset amino acid panel and the remaining 4 from a preset kynurenine panel. Logistic regression was applied to differentiate MDD patients from HCs. **Results:** The best-performing model utilised both feature selection and hyperparameter optimisation and yielded a moderate area under the receiver operating curve (AUC) classification value of 0.76 on the testing data. The top five metabolites identified as potential biomarkers for MDD were 3-hydroxy-kynurenine, valine, kynurenine, glutamic acid, and xanthurenic acid. **Conclusions:** Our study highlights the potential of using an interpretable machine learning analysis model based on amino acids to aid and increase the diagnostic accuracy of MDD in clinical practice.

## 1. Introduction

According to the World Health Organization [1], major depressive disorder (MDD) is a leading cause of disability worldwide, affecting over 280 million people of all ages and nationalities at present. MDD causes 50 million person-years of disability yearly, and depressive disorders are the single largest contributor to non-fatal health loss. With the significant socioeconomic and healthcare burden that MDD imposes, prompt assessment and treatment are essential. Currently, practitioners in the field commonly diagnose MDD in patients by assessing patients’ self-reported symptoms against the designated key symptoms outlined in the fifth edition of the Diagnostic and Statistical Manual of Mental Disorders (DSM-5). Individuals are diagnosed with depression if they report experiencing a depressed mood or loss of interest in activities for at least two weeks in duration, as well as four or more of the other eight symptoms (including weight and sleep disturbances, fatigue, worthlessness, poor concentration, psychomotor disturbances, and suicidal ideation). Practitioners also rely on their professional clinical expertise and past experiences to diagnose, which can be challenging, especially when patients are not forthcoming about their symptoms. These subjective diagnostic methods, coupled with the complex nature of MDD given its wide range of presenting symptoms, have led to the underdiagnosis and inadequate management of the disease.

To date, no definitive biomarkers have been identified for the diagnosis, prognosis, and therapeutic management of MDD [2]. Despite the existence of large-scale genome-wide association studies that aim to identify loci linked to depression, their success in distinguishing and prognosticating the disease remains equivocal [3]. However, advancements in genomics and metabolomics remain at the forefront of psychiatry biomarker discovery, which may, in the future, guide the prognosis and treatment response of patients afflicted with psychiatric disorders [4]. Amino acids, for example, have been steadily gaining traction as potential biomarkers for MDD in recent years [2,5,6,7]. Depression can be attributed to a lack of monoamine neurotransmitters, including serotonin, dopamine, and norepinephrine, according to the well-established monoamine hypothesis. This was substantiated by the fact that antidepressants that target monoamine depletion increase neurotransmitter levels, thus improving the function of the monoaminergic system [8] and, in turn, improving clinical symptoms. In addition, it has been reported that depressed patients have decreased levels of the amino acid precursors tryptophan and tyrosine, from which serotonin and norepinephrine are synthesised [9].

Baranyi et al. [10] showed that branched-chain amino acids (BCAA) isoleucine, leucine, and valine had decreased levels in depressed subjects, with the concentration of these amino acids inversely associated with the Hamilton Depression Rating Scale (HAM-D) scores. Kynurenine, 3-hydroxykynurenine, and kynurenate concentrations were shown to be negatively associated with suicidal ideation, while citrate and alanine were positively associated with suicidal ideation [11]. Amino acids such as GABA were associated with the severity of depression as well [11]. These findings suggest that amino acids do, indeed, harness a predictive potential in depression symptom manifestation, disease progression and predicting treatment response.

A study by Ding and colleagues found that patients with MDD had lower levels of leucine and higher levels of alanine, serine, and proline in their blood serum compared to healthy controls (HCs) [12]. These findings are corroborated by Hung et al., which show that patients with MDD in complete remission presented significantly lower serum levels of metabolites related to pyruvate metabolism when compared to pre-remission levels, which is linked to the metabolism of amino acids such as proline and alanine, amongst others [13]. Further, in a double-blind ketamine and placebo crossover randomised controlled trial involving the participation of unmedicated MDD patients and HCs, the plasma metabolic profiling conducted by Moaddel et al. found that treatment with intravenous ketamine resulted in a slight increase in kynurenine, a metabolite of tryptophan, in the blood serum of patients around 4 h after treatment [14]. Given that low levels of tryptophan and kynurenine are commonly observed in MDD [15,16], these results reinforce the therapeutic role of ketamine in the treatment of MDD [17] and the potential of amino acids to serve as biomarkers for MDD.

Nevertheless, it is important to recognise that the aetiology of MDD is multifactorial, complex, and poorly understood [18,19]. This contributed to the advent of precision psychiatry, which aims to translate research findings into providing individualised clinical care to patients with psychiatric disorders [20]. In the field, multiple data types are often integrated with machine learning and artificial intelligence algorithms, which serve to identify complex patterns from observational datasets [21,22] and leverage such learned insight into estimating predictive outcomes for new, unknown data and/or events in the future [20]. Previous studies incorporating machine learning methods have shown that changes in peripheral amino acid levels can differentiate depressed subjects and HCs with high reliability [5]. In one study, levels of GABA, dopamine, kynurenine, and tyramine accurately differentiated depressed subjects from healthy subjects at approximately 96.8% and 95.3% in the training and testing set, respectively, and separated subjects with unipolar and bipolar depression precisely [23]. With the key role played by biomarker usage based on machine learning and artificial intelligence methods in precision psychiatry [24], the first aim of this study was to demonstrate that combining machine learning with serum amino acid levels can be used to achieve a reasonable prediction performance of MDD diagnosis.

Interpretable machine learning models have the potential to generate knowledge via hypothesis generation [25,26]. Complex machine learning models such as neural networks, support vector machines, and ensemble models can learn complex patterns in data for high predictive performance. However, the disadvantage of such complex models is that they are less interpretable than simpler models. Such simple and interpretable machine learning models can quantify feature importance to generate knowledge about which features were most responsible for guiding the predictive decision of the model, thereby generating new hypotheses [27,28]. Therefore, the second aim of this study was to identify which amino acids are most important in diagnosing MDD using an interpretable machine learning model. The hypothesis for this study is that amino acids, when integrated with machine learning, can enhance the diagnostic accuracy of MDD in a manner that can be easily understood and interpreted.

## 2. Materials and Methods

### 2.1. Sample Size and Participants

For the sample size, reference was made to a study by Pan and colleagues [29], which compared 50 healthy controls and 50 unmedicated MDD patients and reported the effect size of glutamic acid concentration (Cohen’s *d* value of 0.91). For a two-tailed Welch’s *t*-test to achieve 80% power and 0.05 probability of type I errors, assuming a normal distribution, this study derived a minimum number of 26 subjects per group to detect differences in amino acid concentrations between groups. In addition to the limitations of study grant funding, this contributed to the final study sample size of 140.

In total, 140 participants were recruited for this cross-sectional study with 70 MDD patients and 70 HCs matched for sex, age, and ethnicity. All participants were aged between 21 and 50 years, were English-speaking, and right-handed. The patients were recruited from the outpatient psychiatry clinics of a university hospital in Singapore. They were diagnosed with MDD by their psychiatrist following the DSM-5 criteria. Patients with other significant psychiatric disorder comorbidities, such as bipolar depression, schizophrenia, and substance use disorder, were excluded from the study. The HCs were recruited from the community by word-of-mouth and matched with MDD patients accordingly. All subjects were excluded if they had a neurological disorder or medical conditions that could affect the central nervous system. Each subject’s depressive symptoms and disease severity were assessed using the 17-item version of the Hamilton Rating Scale for Depression (HAM-D 17), with scores of 8 to 16 indicating mild depression, 17 to 23 indicating moderate depression, and 24 or higher indicating severe depression.

All study details were fully explained to the participants, and their written, informed consent was obtained. Recruited subjects provided their sociodemographic data, and patients’ clinical information was obtained from them and verified with computer records; all subjects also completed written questionnaires, had their blood drawn during the study visit and had their collected data de-identified. It was difficult to standardise the collection time and lifestyle characteristics, such as the subjects’ nutrition and sleep, as subjects’ samples were collected once they enrolled on the study.

### 2.2. Blood Collection and Metabolite Analysis

Blood sample collection and serum amino acid profiling were conducted using procedures employed in previous studies [27,28]. In total, 21 total metabolites were analysed: 17 from a preset amino acid panel (glycine, alanine, serine, proline, valine, leucine, isoleucine, methionine, histidine, phenylalanine, tyrosine, aspartic acid, glutamic acid, ornithine, citrulline, arginine, and tryptophan) and four from the preset kynurenine panel (kynurenine, kynurenic acid, xanthurenic acid, and 3-Hydroxykynurenine).

### 2.3. Classification Algorithm

Logistic regression was selected because of its interpretability in understanding which features are the most important for the prediction task [5,30,31,32,33,34], compared to more complex models such as deep neural networks [34,35]. This is a crucial characteristic of the logistic regression model for this research, given that the main objective of this paper is to determine which amino acids are most significant for MDD prediction. Furthermore, logistic regression is a well-established algorithm in the literature that differentiates MDD and healthy control subjects using metabolomic data [12,29,36]. L1-regularized (i.e., LASSO-regularized) logistic regression was implemented using the LogisticRegression function from the Scikit-learn library [37].

### 2.4. Cross-Validation Framework

Nested cross-validation was used to avoid an over-optimistic prediction performance and to provide an unbiased estimate for a small sample size while simultaneously allowing for hyperparameter optimisation and feature selection [6,7]. The nested cross-validation method comprises two loops: firstly, the outer loop to assess the prediction performance on unseen data in the test set, and secondly, the inner loop to train the model on the training set and to select the best-performing trained model based on the prediction performance on the validation set. To avoid an over-optimistic prediction performance on the test set, hyperparameter optimisation and feature selection are performed only on data within the inner loop. As such, data within the test set will remain unseen by the trained model.

**Figure 1:**
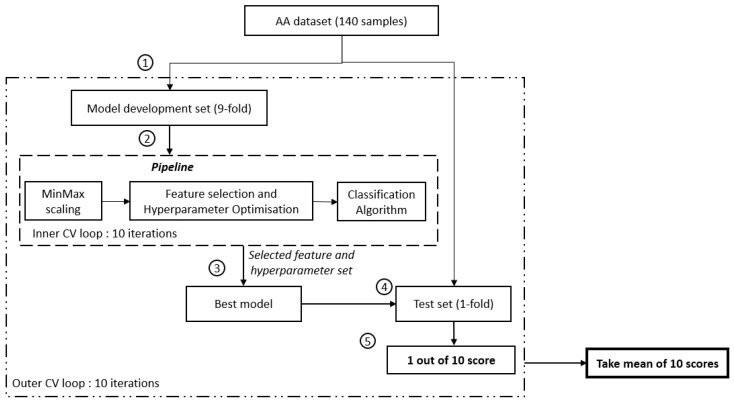
Flowchart of the study. ① Split of the model development and evaluation set, ② model development set will be fed into the pipeline and further search for the best feature set using GridSearchCV. SelectKbest with ANOVA F-value f_classif (min feature = 1, max_feature = 21, step = 1) is used to select the feature, ③ establish the best mode using the selected feature set, ④ evaluate the developed model using an unseen test set, and ⑤ determine the performance score of 1 iteration.

Amino acid data from 140 subjects (70 MDD subjects and 70 HCs) were split into a 9-fold model development set and a 1-fold test set for the outer loop (see Figure 1). The inner loop comprises only data from the 9-fold model development set, split further into a 9-fold training set and a 1-fold validation set. Within the inner loop, minimum-maximum scaling was fit to the training data and subsequently applied to the training and validation data. This normalises feature values to the range [0, 1]. Minimum-maximum scaling was selected to preserve the original distribution shape and thus preserve the information embedded within the data [38,39]. This allows feature selection to be performed later based on the original distribution of the data rather than an altered distribution.

A filter-based feature selection method in the form of a one-way analysis of variance (ANOVA) was performed, whereby the ANOVA F-value was utilised to rank the features. The higher the ANOVA F-value, the more important a feature is deemed to differentiate between the MDD and healthy control groups. Hyperparameter optimisation was implemented. To be clear, the only hyperparameter that was optimised was C, the inverse of L1 regularisation strength for the logistic regression model.

Grid search (via GridSearchCV function from Scikit-learn [37]) was implemented to perform an exhaustive search over both features and C (i.e., the inverse of L1 regularisation strength hyperparameter values). The search range of C spanned 11 values, including [1, 11, 21, 31, 41, 51, 61, 71, 81, 91, 101]. Features were added one at a time, and the prediction performance on the validation set was computed for each combination of features and L1 regularisation value. Once all combinations of features and L1 regularisation values have been computed, this process undergoes 9 more repetitions (10 repetitions in total for each of the 10 folds within the model development set to take turns being the validation set). The combination of features and L1 regularisation value with the highest prediction performance on the validation set is then selected as the best model. The prediction performance of the best model is then evaluated based on the prediction performance of the test data in the outer loop.

The entire process (comprising minimum-maximum scaling, feature selection, hyperparameter optimisation, and the 10 repetitions to obtain the validation set prediction performance) then undergoes 9 more repetitions (10 repetitions in total for each of the 10 folds in the outer loop to take turns being the test set).

### 2.5. Performance Metrics

Four performance metrics are presented in this paper: area under the receiver operating characteristic curve (AUC), accuracy, precision, and recall. AUC was used as the main overall performance metric due to its suitability for medical diagnostic problems. Therefore, the best model was selected based on the AUC prediction performance on the validation set during cross-validation.

## 3. Results

### 3.1. Sample Characteristics

HCs and patients with MDD did not differ significantly in terms of age, sex, or ethnicity (*p* > 0.05; see Table 1).

They did, however, differ in relation to several factors, including years of education, incidence of familial mental illness, history of traumatic experiences, perceived level of social support, and HAM-D 17 scores. MDD patients were more likely to have a history of mental illness in their families (*p* = 0.032) and reported lower social support (*p* < 0.001) when compared to HCs. As anticipated, individuals with MDD obtained significantly higher scores on the HAM-D relative to HCs (*p* < 0.001), with 70% being moderately to severely depressed.

### 3.2. Logistic Regression Model Classification Performance

**Figure 2:**
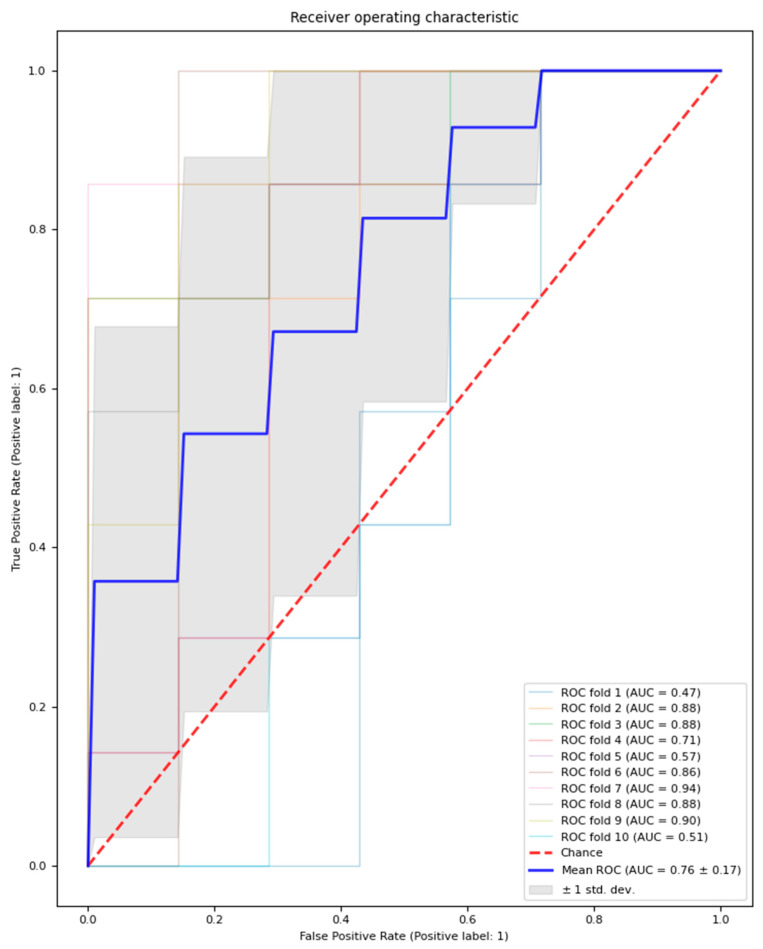
Outer loop AUC result for the model with feature selection and hyperparameter optimisation. Mean AUC results across 10 folds are represented by a bolded blue line; the chance AUC result is represented by a red dotted line.

The logistic regression model with feature selection and hyperparameter optimisation classified MDD patients and healthy controls with an average performance across test sets in the outer loop with an AUC of 0.76 (±0.16), accuracy of 68.6% (±15.7%), precision of 71.2% (±18.7%), and recall of 65.7% (±21.4%). Additionally, an average of 14.6 features (±1.56) were selected across the outer loop. The outer loop AUC result is visualised in Figure 2.

The performance of the logistic regression model with feature selection and hyperparameter optimisation can be compared against two benchmark versions of the logistic regression model. Namely, a version without feature selection but with hyperparameter optimisation and another version without both feature selection and hyperparameter optimisation in Table 2.

Additionally, we evaluated the performance of the logistic regression model with feature selection and hyperparameter optimisation by computing binary classification positive likelihood ratios (LR+) and negative likelihood ratios (LR−). This test was implemented using the class_likelihood_ratios function from Scikit-learn [34] The positive class is the MDD class, and the negative class is the HC class. We note that across the 10 outer folds, there are 2 out of 10 instances where there are 0 false positives. In these two instances, the LR+ was infinitely high, undefined, and returned as a NaN (not a number) value. However, to report a mean non-NaN LR+ value, these two instances with zero false positives are ignored, and the mean is taken across the remaining eight outer folds. The mean LR+ across the remaining 8 non-NaN outer folds was 2.37, and the mean LR− across the 10 outer folds was 0.49.

### 3.3. Logistic Regression Model-Selected Features

**Figure 3:**
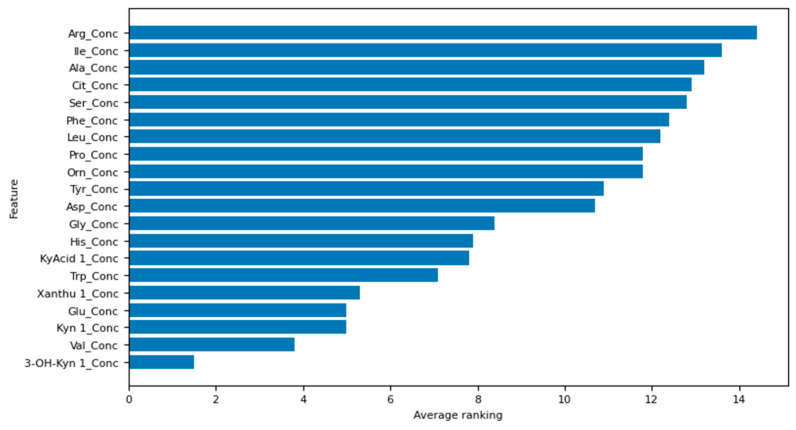
Feature selection results for the model with both feature selection and hyperparameter optimisation.

The top five amino acids identified by our best-performing logistic regression model (the model with both feature selection and hyperparameter optimisation) in order of decreasing ranking are as follows: 3-hydroxy-kynurenine, valine, kynurenine, glutamic acid, and xanthurenic acid. The ranking of all the amino acids used in this study can be interpreted from Figure 3, whereby the lower the average ranking, the more important the amino acid.

## 4. Discussion

In this study, depressed patients were found to have significantly lower levels of education, were more likely to have a family history of mental illness, experienced trauma and reported lower social support than HCs, which concurred with findings from the existing literature. Research has shown that lower education levels are correlated with diminished mental well-being and an increased susceptibility to the development of psychiatric disorders [40]. A positive family history of depression is associated with a personal history of depression, and it is the most significant risk factor for developing a depressive disorder [41]. Traumatic experiences are also associated with an increased risk for MDD [42]. Individuals who perceived less social support were at a greater risk for developing depressive symptoms [43].

While many relevant works implement traditional statistical techniques to differentiate MDD and healthy control subjects [44,45,46,47,48], there are substantially fewer studies on using amino acid data in machine learning for the predictive classification of MDD and HCs. Despite many machine learning studies aimed at distinguishing between individuals with MDD and HCs, effectively applying machine learning techniques to diagnose MDD in the clinical setting still poses significant challenges. This difficulty could be explained by the fact that conventional statistical methods excel at detecting relationships at the population level. In contrast, machine learning is adept at identifying generalisable patterns in the data, enabling predictions at the individual level [49]. In our study, which deals with data from multiple amino acids, machine learning techniques were generally considered more suitable due to their ability to discern complex relationships between multiple variables [50].

In this study, the logistic regression model with feature selection and hyperparameter optimisation classified MDD and healthy control subjects with an average performance across test sets in the outer loop with an AUC of 0.759. A study conducted by Hung et al. [13], which also aimed to develop a predictive model to discriminate between MDD patients and HCs with as high a performance score as possible, achieved a test set performance AUC of 0.784 using the linear support vector machine (SVM) classifier to discriminate between MDD patients and HCs. This disparity in model performances could be attributed to the utilisation of the logistic regression machine learning model in this study, whose secondary aim was to construct an amino acid feature importance ranking. Hence, it is unsurprising that a simpler logistic regression model would slightly underperform the more complex linear SVM model. Furthermore, it is worth mentioning that this study includes a more comprehensive set of 21 amino acids, while Hung et al.’s study focused only on 8 metabolites.

Another related study by Zheng et al. [23] achieved an outstanding test set performance AUC of 0.96 using the least-squares SVM model. While amino acids were used as part of the input data for the model in Zheng et al.’s study, they did not exclusively focus on amino acids; other metabolites, such as glucose and lipids, were also included. This could partly explain the substantial discrepancy in model performance between Zheng et al.’s model and the mode proposed by our study. Furthermore, Zheng et al.’s study included a small sample size of 126 subjects; however, it had a single split of the training and test sets. At such small sample sizes (like this study’s sample size), it is crucial to note that a single random training-test set split may lead to highly over-optimistic results. Therefore, this could also partly explain the difference in model performances between this study and Zheng et al.’s study. Also, like Hung et al.’s study, Zheng et al.’s study did not include any feature importance rankings of metabolites.

The third related study by Liu et al. [51] was able to identify blood metabolite markers which distinguished melancholic depressed patients (*n* = 90) from HCs (*n* = 97) with 80% accuracy after employing the ensemble feature selection framework (EFSF). Liu et al. analysed 228 metabolites (of which a union of 76 metabolites from the four feature selection methods was used as input). Of the 76 metabolites, 56 were mapped to the Encyclopedia of Genes and Genomes (KEGG). 11 of the 56 metabolites analysed were amino acids, and 48 of 56 metabolites were increased in melancholic depressed patients versus HCs. While Liu et al. could discriminate between HCs and depressed subjects at a higher rate of 80%, they used a more complex machine learning model known as the ensemble feature selection framework (EFSF), which affected how interpretable the model was.

Regarding this study’s amino acid and metabolite ranking, the top five were determined to be 3-hydroxy-kynurenine, valine, kynurenine, glutamic acid, and xanthurenic acid. These compounds are implicated in neurobiological mechanisms that potentially contribute to the development of depression, rendering them clinically significant. In depression, the kynurenine pathway is thought to be activated, and available tryptophan is diverted away from serotonin production and into further degradation, leading to an increase in the production of 3-hydroxykynurenine (3-HK). 3-HK is considered neurotoxic due to its ability to generate reactive oxygen species, alter mitochondrial function, and induce DNA damage [52], which may be associated with depression [53]. 3-HK is then converted to xanthurenic acid by the enzyme kynurenine aminotransferase, and increased xanthurenic acid levels have also been found in depressed patients relative to controls [54]. Glutamic acid may contribute to depression through its action as an N-methyl-D-aspartate (NMDA) receptor agonist [55], with hypofunctioning NMDA receptors causing cognitive deficits and excessive activation causing excitotoxicity and neurodegeneration [56]. The blood serum concentration of valine has been found to increase in MDD patients and has been shown to be highly correlated with the other BCAAs associated with depression [10,57,58]. It has also been suggested that increased blood serum valine concentration can produce depressive symptoms by decreasing brain serotonin (5-HT) function; it inhibits the transport of 5-HT precursor, L-tryptophan, across the blood-brain barrier [59].

Our study contributes to the limited body of literature on the feasibility of using amino acids as biomarkers for MDD. Given the observed changes in amino acid concentrations and their correlations with MDD, our results have shown that the logistic regression model incorporating feature selection and hyperparameter optimisation can differentiate between MDD patients and HCs with reasonable accuracy. This highlights another main strength of our study, which utilises machine learning to identify specific amino acids that are of significance in the context of MDD.

However, it would be important to acknowledge that the clinical implications of our findings are limited by our study’s relatively small sample size. Furthermore, since our study population consisted mostly of Asian individuals with a mean age of approximately 27 years and most of the patients were receiving prescribed medication, it is unlikely that our findings can be extrapolated to the wider population. Further studies should focus on recruiting a larger sample with different age groups who are unmedicated. In addition, not all MDD patients in our study are medication naive, so our findings could be attributed to treatment effects. Pertinently, combining multimodal data for classification can overcome the limitations of using only a single data source. Therefore, future investigations integrating clinical, omics (including genetic, transcriptomic and proteomic), and neuroimaging data would be crucial for enhancing the diagnostic utility of amino acids as biomarkers of MDD in clinical practice. An assumption of the logistic regression model is the absence of perfect multicollinearity among features [60]. Figure S1 shows the pairwise Pearson correlation coefficient value between pairs of amino acid features used to train the logistic regression model. We note that only 1 out of 210 pairwise entries (%) of the correlation matrix lower triangle was a close-to-perfect correlation (i.e., r equal to or greater than 0.95). Therefore, most features did not have a close-to-perfect correlation with one another, and we consider this assumption to be largely held. This pairwise correlation of 0.96 was between isoleucine and leucine concentrations. Isoleucine and leucine were retained as features because they contain biological significance in MDD patients, as shown by Baranyi and colleagues [10]. Hence, it is unclear if isoleucine or leucine should be removed. Additionally, the mean test AUC performance (across 10 outer folds) numerically declined when the logistic regression model (with feature selection and hyperparameter optimisation) either dropped isoleucine concentration or leucine concentration features, from 0.76 ± 0.17 to 0.73 ± 0.17, and 0.73 ± 0.17, respectively (Table S1). Consequently, including both isoleucine and leucine concentration features leads to a more robust prediction performance.

In summary, our study highlights the potential of using amino acid-based machine learning analysis to predict and diagnose MDD due to its acceptable accuracy, interpretability, and stability. This approach can potentially enhance the reliability of MDD diagnosis in clinical practice, hence warranting further research in this area.

## Figures and Tables

**Table 1:** Demographic and clinical characteristics of subjects.

	MDD (*n* = 70)	HC (*n* = 70)	*p* Value *
Age (years)	28.3 (SD 7.2)	28.2 (SD 7.3)	0.926
Sex			1.000
Male	16 (22.9%)	16 (22.9%)	
Female	54 (77.1%)	54 (77.1%)	
Ethnicity			1.000
Chinese	45 (64.3%)	45 (64.3%)	
Malay	15 (21.4%)	15 (21.4%)	
Indian	9 (12.9%)	9 (12.9%)	
Eurasian	1 (1.4%)	1 (1.4%)	
Education (years)	14.5 (SD 1.8)	15.6 (SD 1.2)	**<0.001**
Perceived social support			
Poor	17 (24.3%)	0 (0.0%)	**<0.001**
Average	44 (62.9%)	18 (25.7%)	
Good	9 (12.9%)	52 (74.3%)	
HAM-D 17 score	19.8 (SD 5.4)	1.9 (SD 2.5)	**<0.001**
Mild (8–16)	21 (30.0%)	4 (5.7%)	
Moderate (17–23)	30 (42.9%)	0	
Severe (≥24)	19 (27.1%)	0	
Family psychiatric history	30 (42.9%)	17 (24.3%)	**0.032**
History of trauma	35 (50%)	14 (20.0%)	**<0.001**
Past admission to a psychiatric ward	16 (22.9%)		
Past suicide attempt	32 (45.7%)		
Pharmacotherapy	60 (85.7%)		

* *p*-values ≤ 0.05 are in bold.

**Table 2:** Validation and test set performance results for three variations of logistic regression models: with feature selection, without feature selection, without feature selection and hyperparameter optimisation.

	Validation Set Performance	Test Set Performance				
Type of Logistic Regression Model	AUC	AUC	Accuracy	Precision	Recall	Number of Features Selected
With feature selection and with hyperparameter optimisation	0.74 ± 0.03	0.76 ± 0.16	68.6 ± 15.7	71.2 ± 18.7	65.7 ± 21.4	14.6 ± 1.56
No feature selection and with hyperparameter optimisation	0.73 ± 0.03	0.72 ± 0.17	67.9 ± 14.0	70.6 ± 17.3	65.7 ± 19.4	21.0 ± 0.00
No feature selection and no hyperparameter optimisation	0.71 ± 0.04	0.73 ± 0.17	65.0 ± 14.8	66.6 ± 20.1	60.0 ± 20.0	21.0 ± 0.00

## Data Availability

The data supporting this study’s findings are available upon request from co-first author Cyrus Su Hui Ho.

## Supplementary Materials

The following supporting information can be downloaded at: https://www.mdpi.com/article/10.3390/jcm13051222/s1, Figure S1: Pearson correlation coefficient (r) results between pairs of amino acid concentration features. Table S1: Validation and test set performance results for three variations of logistic regression models (all 3 with feature selection and with hyperparameter optimisation): original model, model without isoleucine concentration feature, and model without leucine concentration feature.
