# Supplementary material for: Using an Interpretable Amino Acid-Based Machine Learning Method to Enhance the Diagnosis of Major Depressive Disorder
Source: J Clin Med. 2024 Feb 21;13(5):1222. doi: 10.3390/jcm13051222 (PMC10931723; doi:10.3390/jcm13051222)
Supplement: Supplementary file 1 [file jcm-13-01222-s001.zip › jcm-2835009-supplementary.pdf]

Journal of
Clinical Medicine

MDPI

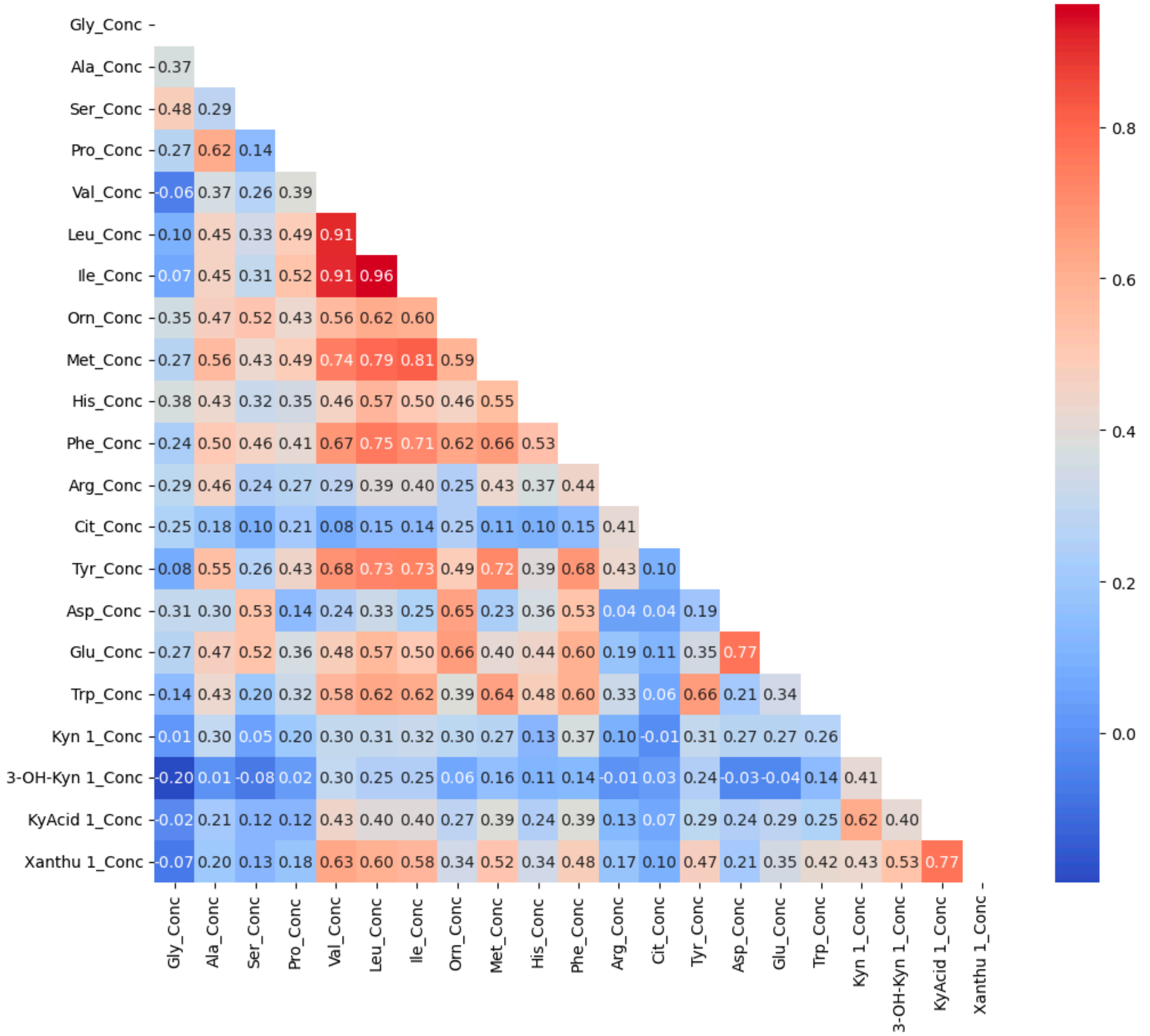

Figure S1. Pearson correlation coefficient (r) results between pairs of amino acid concentration features. Each cell represents r between each unique pair of amino acids. The darker the shade of red of the cell, the more positive the r. The darker the shade of blue of the cell, the more negative the r.

Table S1. Validation and test set performance results for three variations of logistic regression models (all 3 with feature selection and with hyperparameter optimisation): original model, model without isoleucine concentration feature, and model without leucine concentration feature.

	Validation set performance	Test set performance				
**Logistic regression model (with feature selection and with hyperparameter optimisation)**	**AUC**	**AUC**	**Accuracy**	**Precision**	**Recall**	**Number of features selected**
**Original model (with isoleucine and with leucine)**	0.74 ± 0.03	0.76 ± 0.16	68.6 ± 15.7	71.2 ± 18.7	65.7 ± 21.4	14.6 ± 1.56
**Dropped isoleucine**	0.74 ± 0.03	0.73 ± 0.17	66.4 ± 13.9	68.8 ± 18.1	65.7 ± 19.4	14.4 ± 1.86
**Dropped leucine**	0.74 ± 0.03	0.73 ± 0.17	67.1 ± 14.7	70.1 ± 18.9	65.7 ± 20.4	13.9 ± 1.22
